# Assessment of Local Mosquito Species Incriminates *Aedes aegypti* as the Potential Vector of Zika Virus in Australia

**DOI:** 10.1371/journal.pntd.0004959

**Published:** 2016-09-19

**Authors:** Sonja Hall-Mendelin, Alyssa T. Pyke, Peter R. Moore, Ian M. Mackay, Jamie L. McMahon, Scott A. Ritchie, Carmel T. Taylor, Frederick A.J. Moore, Andrew F. van den Hurk

**Affiliations:** 1 Public Health Virology, Forensic and Scientific Services, Department of Health, Queensland Government, Coopers Plains, Australia; 2 College of Public Health, Medical and Veterinary Sciences, James Cook University, Cairns, Australia; 3 Australian Institute of Tropical Health and Medicine, James Cook University, Cairns, Australia; Fundaçao Oswaldo Cruz, BRAZIL

## Abstract

**Background:**

Within the last 10 years Zika virus (ZIKV) has caused unprecedented epidemics of human disease in the nations and territories of the western Pacific and South America, and continues to escalate in both endemic and non-endemic regions. We evaluated the vector competence of Australian mosquitoes for ZIKV to assess their potential role in virus transmission.

**Methodology/Principal Findings:**

Mosquitoes were exposed to infectious blood meals containing the prototype African ZIKV strain. After 14 days incubation at 28°C and high relative humidity, infection, dissemination and transmission rates were assessed. Infection in *Culex annulirostris* and *Cx*. *sitiens* could not be detected. 8% of *Cx*. *quinquefasciatus* were infected, but the virus did not disseminate in this species. Despite having infection rates > 50%, *Aedes notoscriptus* and *Ae*. *vigilax* did not transmit ZIKV. In contrast, *Ae*. *aegypti* had infection and transmission rates of 57% and 27%, respectively. In susceptibility trials, the virus dose required to infect 50% (ID_50_) of *Ae*. *aegypti* was10^6.4^ tissue culture infectious dose_50_ (TCID_50_)/mL. Additionally, a threshold viral load within the mosquito of at least 10^5.1^ TCID_50_ equivalents/mL had to be reached before virus transmission occurred.

**Conclusions/Significance:**

We confirmed *Ae*. *aegypti* to be the most likely mosquito vector of ZIKV in Australia, although the restricted distribution of this species will limit the receptive zone to northern Queensland where this species occurs. Importantly, the role in ZIKV transmission of *Culex* and other *Aedes* spp. tested will be negligible. Despite being the implicated vector, the relatively high ID_50_ and need for a high titer disseminated infection in *Ae*. *aegypti* suggest that high mosquito population densities will be required to facilitate epidemic ZIKV transmission among the currently immunologically naïve human population in Australia.

## Author Summary

Zika virus was first isolated in Uganda in 1947 and exists in a transmission cycle between mosquitoes and non-human primates or humans. Whilst most clinical infections result in a self-limiting febrile illness, Zika virus has recently been linked to neurological syndromes, such as Guillain-Barré syndrome and congenital birth defects. Since 2007, Zika virus has undergone a dramatic range expansion, causing epidemics in nations and territories of the western Pacific and South America. To assess the emergence and transmission risk of Zika virus emerging in Australia, we evaluated the ability of local mosquitoes to become infected with and transmit the prototype African Zika virus strain. In agreement with its substantiated role in Zika virus transmission overseas, Australian *Aedes aegypti* were shown to be competent vectors. Coupled with its anthropophilic feeding behavior, this species should be considered the primary potential Zika virus vector in Australia. Although other common Australian species, such as *Ae*. *notoscriptus* and *Ae*. *vigilax*, were readily infected, they did not transmit the virus. The species of *Culex* tested were either refractory to infection or had a low infection rate. We also demonstrated that the Zika virus dose necessary to infect *Ae*. *aegypti* was higher than virus levels reported in infected humans. Finally, a high threshold level of virus circulating through the mosquito body was required before *Ae*. *aegypti* transmitted the virus. These results suggest that an outbreak of Zika virus in Australia would require high mosquito population densities and a susceptible human population.

## Introduction

Zika virus (ZIKV) was first isolated from a rhesus monkey in the Zika Forest of Uganda in 1947 during studies investigating the ecology of yellow fever virus [1]. It was subsequently isolated from *Aedes africanus*, indicating a mosquito-borne transmission cycle. It was not until almost 20 years later that human clinical disease attributed to ZIKV infection was recognized [2]. ZIKV circulates in a sylvatic transmission cycle between non-human primates and mosquitoes. Humans are only incidentally infected in this sylvatic cycle, but are the primary amplifying hosts during epidemics [3, 4]. Although 80% of infections remain asymptomatic, clinical disease caused by ZIKV is typical of many mosquito-borne viruses, and is characterized by fever, muscle and joint pain, headache, conjunctivitis, gastrointestinal manifestations and rash [2, 5, 6]. However, during its rapid expansion in the last 5 years, more severe disease manifestations have been recognized among those with Zika virus–like disease, most notably neurological symptoms, including Guillain-Barré syndrome, microcephaly and other central nervous system malformation in neonates [7, 8].

Based on serological evidence, ZIKV infection of humans has historically been restricted to Africa and Asia [9]. In 2007, an outbreak of ZIKV occurred on Yap Island in the western Pacific Ocean [5], signaling the beginning of an unprecedented range expansion of this virus. Since 2013, ZIKV has affected a number of countries and territories in the western Pacific, resulting in outbreaks of hundreds or thousands of suspected cases [10]. The 2013 epidemic in French Polynesia was the largest ever reported up to that point and resulted in an estimated 32,000 cases, representing 11.5% of the population [11]. In 2014 the virus was introduced into Brazil, from where it is believed to have subsequently spread to a number of countries in South America, causing over a million suspected cases [10, 12]. Between 2007 and June 2 2016, ZIKV was recognized in 63 countries or territories [13].

The risk of ZIKV spread to Australia is very high due to its close geographical proximity to the epidemic region in the western Pacific and high intake of travelers from this area. Indeed, between 2012 and June 3 2016, 60 people infected with ZIKV have traveled to Australia [14]. Should a viremic patient be bitten by a local mosquito, or transmit the virus sexually [15], there is potential for autochthonous transmission of the virus to occur, but this has not been reported to date. Clinical similarity of ZIKV disease with that caused by arboviruses, such as dengue (DENV) and chikungunya (CHIKV), could delay ZIKV identification or detection of transmission, and there are currently no established vaccines or therapeutics. Therefore, it is very important to estimate the risk of ZIKV establishment in Australia for the purposes of heightened public awareness, and for adequate and appropriate response of public health authorities.

In the current study we evaluated the ability of common Australian mosquito species to become infected with and transmit the African lineage of ZIKV. Although the Asian lineage is responsible for the recent activity in the Pacific and South America, we were not able to obtain an isolate to facilitate the assessment of the vector competence of local mosquito fauna for this ZIKV lineage in a manner that was timely for formulation of targeted control strategies. The sylvan vectors circulating ZIKV between primates in Africa are tree-hole inhabiting *Aedes* spp., of which *Ae*. *africanus* is the most important [4]. The primary urban vectors are *Ae*. *aegypti* and *Ae*. *albopictus* [9]. Of the species implicated abroad, only *Ae*. *aegypti* occurs on the Australian mainland, albeit with a distribution restricted to northern Queensland [16]. *Ae*. *albopictus* is currently restricted to the Torres Strait islands off northern Australia [17] and was not tested in the current experiments. In terms of other potential vectors, Australia has a number of other species that could potentially transmit ZIKV. For instance, *Ae*. *notoscriptus* is a widespread urban species throughout Australia and was shown to be a competent laboratory vector of yellow fever virus [18]. Another potential species is *Ae*. *vigilax*, which has a widespread coastal distribution, is a notorious biter and a highly competent laboratory vector of CHIKV [19]. Finally, it has been postulated that *Culex* spp., most notably *Cx*. *quinquefasciatus*, may play a role in transmission in South America [20].

## Materials and Methods

### Mosquitoes

Mosquitoes were collected as eggs or adults from several locations in Queensland. Adult *Ae*. *vigilax*, *Ae*. *procax*, *Cx*. *annulirostris* and *Cx*. *sitiens* were collected using Centers for Disease Control light traps (Model 512, John Hock Co., Gainesville, FL) baited with CO_2_ (1kg dry ice) from several suburbs in Brisbane, southeastern Queensland. Adult mosquitoes were transported to the laboratory and exposed to ZIKV within 5 h of collection.

The *Ae*. *aegypti* used were F_4_ generation females from eggs collected using ovitraps from Townsville, northern Queensland in March 2015. Eggs of *Ae*. *notoscriptus* and *Cx*. *quinquefasciatus* were collected using ovitraps and infusion buckets, respectively, from several suburbs in Brisbane. All larvae were reared at 26°C and 12:12 L:D. Both *Aedes* spp. were fed Hikari Cichlid Staple pellets (Kyorin Co. Ltd, Himeji, Japan). First and second instar larvae of *Cx*. *quinquefasciatus* were fed a 1:1 mixture of brewer’s yeast (Brewer’s Yeast, Healthy Life) and fish flakes (Wardley’s Tropical Fish Food Flakes, The Hartz Mountain Corporation, NJ), whilst third and fourth instars were fed Hikari Cichlid Staple pellets (Kyorin co. Ltd, Himeji, Japan). Adults were held for 3–7 d at 26°C 12:12 L:D, and high relative humidity, and fed on 15% honey water *ad libitum*. Mosquitoes were starved for 24 h prior to virus exposure.

### Virus strain

The ZIKV strain (MR 766) was the prototype strain isolated from a rhesus macaque monkey in the Zika Forest, Uganda in 1947. The virus was sourced from the American Type Culture Collection (Manassas, VA, USA). It had been passaged 146 times in adult mouse brain, once in suckling mouse brain and three times in in African green monkey kidney (Vero) cells.

### Exposure of mosquitoes to ZIKV

Mosquitoes were allowed to feed for 2 h on an infectious blood meal containing stock virus diluted in commercially available defibrinated sheep blood (Institute of Medical and Veterinary Science, Adelaide, Australia). The blood meal was housed within a Hemotek feeding apparatus (Discovery Workshops, Accrington, Lancashire, UK) that was fitted with pig intestine as the membrane. Due to sufficient numbers and relatively high feeding rates, *Ae*. *aegypti* and *Ae*. *notoscriptus* were exposed to serial 10-fold dilutions of ZIKV, with 10^6.7± 0.2^ tissue culture infectious dose_50_ (TCID_50_)/mL the highest dose. The latter species was exposed to an additional blood feed at the highest dose. Due to limited numbers and low feeding rates, the other species were only exposed to blood meals containing a virus titer of 10^6.7± 0.2^ TCID_50_/mL. Pre- and post- feeding samples of the blood/virus mixture were diluted 1:10 in growth media (GM; Opti-MEM (Gibco, Invitrogen Corporation, Grand Island, NY) containing 3% foetal bovine serum (FBS), antibiotics and antimycotics), and stored at -80°C.

Immediately following virus exposure, mosquitoes were anesthetized with CO_2_ and engorged mosquitoes were placed in 900 ml gauze-covered containers. All mosquitoes were maintained on 15% honey water at 28°C, high relative humidity and 12L:12D light cycle within an environmental growth cabinet.

### Assessment of infection, dissemination and transmission

Mosquitoes were processed at day 14 post-exposure. Due to sufficient numbers, *Ae*. *aegypti* were also processed at 5, 7 and 10 d post exposure, whilst the additional *Ae*. *notoscriptus* were processed at day 7 post exposure. The ability for mosquitoes to become infected with and transmit ZIKV was assessed using a modified *in vitro* capillary tube technique [21]. Briefly, mosquitoes were anesthetized with CO_2_, and the legs and wings removed. The saliva was collected by inserting the proboscis of the mosquito into a capillary tube containing GM with 20% FBS. After 30 min, the contents of the capillary tube were expelled into 500 μl of GM with 3% FBS. The legs+wings and bodies were placed in separate 2 mL tubes containing 1 mL of GM with 3% FBS and a single 5 mm stainless steel bead. Detection of virus in the legs+wings indicates that the mosquito has developed an infection whereby the virus has escaped the midgut and disseminated throughout the hemocoel, thus bypassing the midgut escape barrier [22]. All samples were stored at -80°C.

### Virus assays

#### Titration of initial blood/virus mixtures

Blood/virus mixtures were titrated as serial ten-fold dilutions in 96 well microtiter plates seeded with confluent monolayers of C6/36 cells. Plates were incubated at 28°C for 10 d before being fixed in PBS/acetone and stored at -20°C. Infection with ZIKV was detected using a cell culture enzyme immunoassay (CC-EIA; [23]) and the pan-flavivirus reactive monoclonal antibody 4G2 (provided by Roy Hall, University of Queensland, Australia).

#### Detection of ZIKV in mosquito components and saliva expectorates

Bodies and legs+wings were homogenized separately using a QIAGEN TissueLyser II (Qiagen, Hilden, Germany) and centrifuged at 14,000g for 5 min. Viral RNA was extracted from 140 μl of each homogenate and the saliva expectorates using the Qiagen BioRobot Universal System and QIAamp Virus BioRobot MDx Kit (Qiagen, Clifton Hill, Australia). All samples were analyzed for ZIKV RNA using a real-time TaqMan RT-PCR designed in the nonstructural protein 5 (NS5) gene. Primer and dual-labeled probe sequences (genome nucleotide positions corresponding to ZIKV MR766, GenBank accession number AY632535) were as follows: forward primer (Zika –F-2007) 5’-^9845^CCTCAAGGATGGGAGATCCA^9864^-3’, reverse primer (Zika-R-2007) 5’-^9908^ AGCTCGGCCAATCAGTTCAT^9889^-3’ and probe (Zika-FAM-2007) 5’FAM-^9868^ TGGTCCCTTGCCGCCACCA^9886^ TAMRA-3’. Primer and probe oligonucleotides were synthesized by Sigma-Aldrich (Australia). Separate synthetic primer and probe oligonucleotides were also designed to prepare *in vitro* transcribed RNA positive assay controls. The synthetic assay control sequences incorporated T7 promoter (bold), ZIKV TaqMan primer or probe (italics) and ubiquitin-conjugating enyzyme E2 D2 (UBE2D2) housekeeping gene (underlined) sequences (protocol adapted from Smith et al. [24]). The primer assay control (NS5-Zika-synPri) 5’-AAAA**TAATACGACTCACTATA**GGG *CCTCAAGGATGGGAGATCCA*ATGATCTGGCACGGGACCCTCCAA *ATGAACTGATTGGCCGAGCT*-3’ and probe assay control (NS5-Zika-synPro) 5’-AAAA**TAATACGACTCACTATA**GGGTGAAGAGAATCCACAAGGAATTGAA *TGGTCCCTTGCCGCCACCA*ACAGTGTTCAGCAGGTCCTGTTG-3’ synthetic assay control oligonucleotides were synthesized by GeneWorks Pty. Ltd. (Adelaide, Australia).

*In vitro* RNA transcription of 2–5μg of each synthetic assay control was performed for one hour at 37°C in a 100μl volume using the Riboprobe System-T7 kit (Promega, United States). Each RNA preparation was subjected to DNase treatment, DNase inactivation (TURBO DNA-free kit, Life Technologies, Unites States) and RNA purification using a spin column, according to the manufacturer’s instructions (RNeasy Mini Kit, QIAGEN, Australia). The DNase treatment, inactivation and RNA purification process was conducted twice to ensure sufficient reduction of amplifiable template DNA.

The ZIKV-NS5 real-time RT-PCR was performed using a Rotor-Gene 6000 real-time PCR machine (QIAGEN, Australia). Amplification of ZIKV RNA and detection of the 64 bp product took place in a 20 μL single-tube, Superscript III Platinum one-step qRT-PCR (Invitrogen, Carlsbad, CA). Reactions contained 0.4 μL Superscript III RT/Platinum *Taq* mix, 10.0 μL of 2X reaction mix, 650 nM primers, 250 nM dual-labeled probe, 50 nM ROX reference dye, and 5 μL of extracted sample RNA or serially diluted extracted ZIKV MR 766 RNA. Each amplification run included positive primer control (5 μl synthetic primer assay control RNA) and probe control (5 μl synthetic probe assay control RNA) reactions as previously described [24]. The cycling conditions consisted of one cycle at 50°C for 5 min, one cycle at 95°C for 2 min, and 40 cycles at 95°C for 3 s and 60°C for 30 s. The threshold cycle number (*C*_t_) was determined for each sample and a *C*_t_ value ≥40 cycles was chosen to indicate no RNA detection.

The ZIKV stock was titrated in 96 well microtiter plates seeded with Vero African green monkey cells (ATCC, CCL-81) and the CC-EIA was used to determine TCID_50_/mL values [25]. Viral RNA was extracted as above and ten-fold dilutions were used to generate standard curves for the ZIKV-NS5 real-time RT-PCR. Each dilution (10^−4^–10^−9^) was run in duplicate and presented as a mean using Rotor-Gene software. Standard curves were plotted as *C*_t_ values versus –log TCID_50_/mL and were used to calculate ZIKV TCID_50_ equivalents/mL in each sample (S1 Fig).

### Statistical analysis

The susceptibility of *Ae*. *aegypti and Ae*. *notoscriptus* to infection with ZIKV was calculated by probit analysis using SPSS release 16.0.0. Log-log models were assessed using the Pearson χ^2^ goodness-of-fit statistic and susceptibility to infection was expressed as ID_50_ ± 95% confidence intervals (CIs) and defined as the virus dose per mL at which 50% of mosquitoes tested positive for ZIKV infection in the TaqMan RT-PCR. Infection, dissemination and transmission rates between *Ae*. *aegypti* and the other species were analyzed using Fisher’s Exact Tests with two-tailed *P*-values (GraphPad Prism Version 6). ZIKV TCID_50_/mL equivalents were tested for differences between species, and within *Ae*. *aegypti* using the Kruskal-Wallis test (GraphPad Prism Version 6).

## Results

### Susceptibility of *Ae*. *aegypti* and *Ae*. *notoscriptus* to ZIKV infection

**Fig 1:**
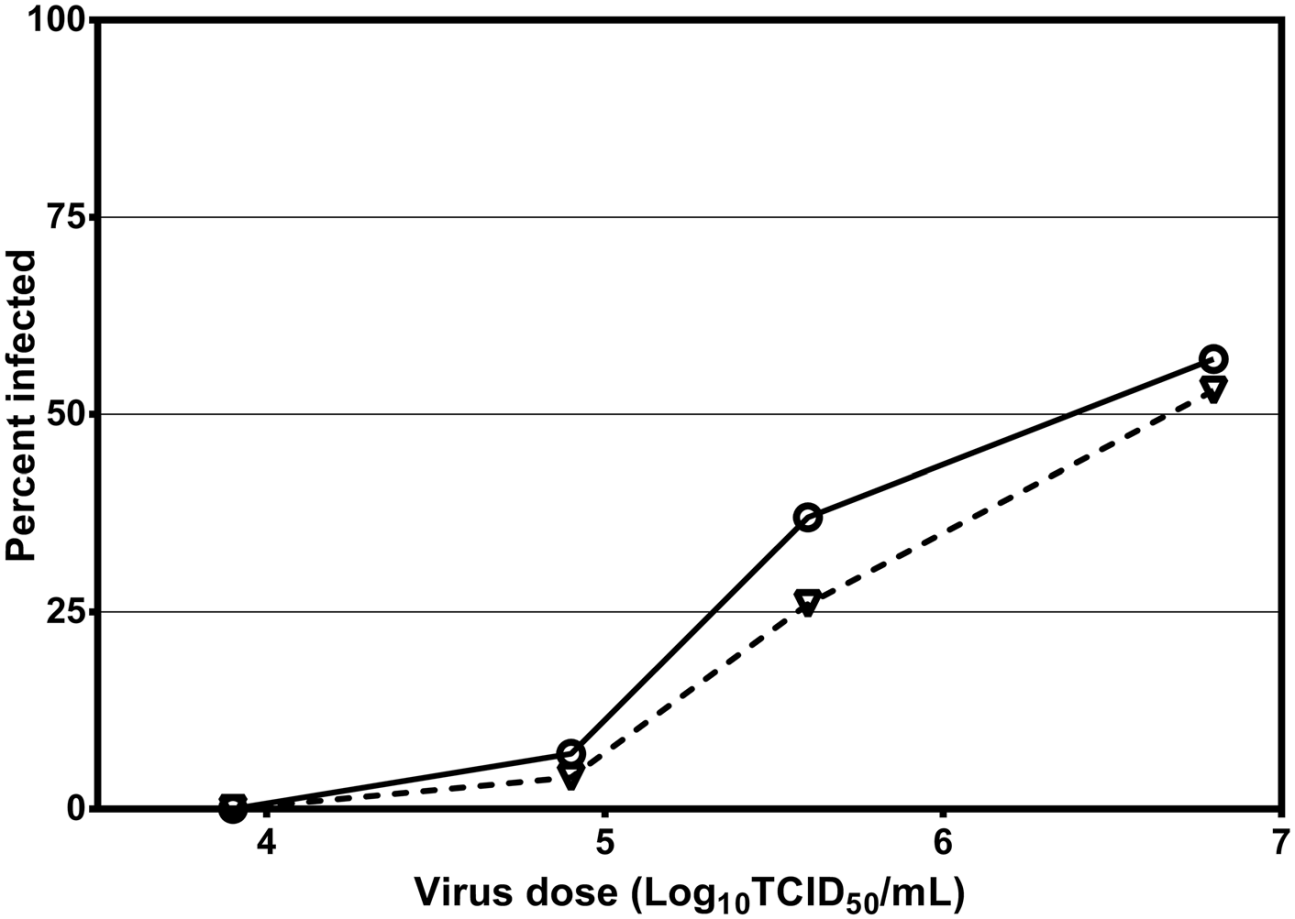
Susceptibility of *Ae*. *aegypti* and *Ae*. *notoscriptus* to ZIKV infection. Percent infection rates in *Ae*. *aegypti* (circles) and *Ae*. *notoscriptus* (triangles) exposed to serial dilutions of ZIKV and tested at 14 d post-exposure.

To assess their susceptibility to infection, *Ae*. *aegypti* and *Ae*. *notoscriptus* were exposed to ZIKV doses ranging from 10^3.9^ to 10^6.8^ TCID_50_/mL and their bodies tested for infection 14 d post exposure (Fig 1). Both species were susceptible to infection, with ID_50_s of 10^6.4^ (10^6.0^ and 10^7.1^, 95% CL) TCID_50_/mL (χ^2^ = 2.49, df = 2, P = 0.288) and 10^6.6^ (10^6.2^ and 10^7.4^, 95% CL) TCID_50_/mL (χ^2^ = 8.49, df = 2, P = 0.654) for *Ae*. *aegypti* and *Ae*. *notoscriptus*, respectively.

### Infection, dissemination and transmission rates in Australian mosquitoes

Five out of seven species, including all species of *Aedes*, were infected 14 days after being exposed to blood meals containing 10^6.7 ± 0.2^ TCID_50_/mL of ZIKV (Table 1). Out of the 3 *Culex* spp. tested, *Cx*. *annulirostris* and *Cx*. *sitiens* were refractory to infection and only 2 out of 30 *Cx*. *quinquefasciatus* were infected but none developed a disseminated infection. On day 14 post exposure, infection rates in *Ae*. *notoscriptus*, *Ae*. *procax* and *Ae*. *vigilax* did not significantly differ (*P* > 0.05) from *Ae*. *aegypti*. With the exception of one cohort of *Ae*. *notoscriptus*, dissemination rates in these three species did not significantly differ (*P* > 0.05) from *Ae*. *aegypti*. However, *Ae*. *aegypti* was the only species that was able to transmit ZIKV, with transmission first observed at day 10 post exposure.

**Table 1:** Infection, dissemination and transmission rates in seven Australian mosquito species exposed to a blood meal containing 10^6.7 ± 0.2^ TCID_50_/mL of ZIKV.

Species	Day PE	% infection^a^		% dissemination^b^		% dissemination/ infection^c^		% transmission^d^		% transmission/ dissemination^e^	
*Ae*. *aegypti*	5	40	(10/25)	8	(2/25)	20	(2/10)	0	(0/25)	0	(0/0)
	7	52	(13/25)	36	(9/25)	69	(9/13)	0	(0/25)	0	(0/0)
	10	40	(10/25)	28	(7/25)	70	(7/10)	12	(3/25)	43	(3/7)
	14	57	(17/30)	40	(12/30)	71	(12/17)	27	(8/30)	67	(8/12)
*Ae*. *notoscriptus*	7	72	(18/25)	8	(2/25)*	11	(2/18)*	0	(0/25)	0	(0/0)
	14	60	(18/30)	0	(0/30)*	0	(0/0)	0	(0/30)*	0	(0/0)
	14	53	(16/30)	20	(6/30)	38	(6/16)	0	(0/30)*	0	(0/0)
*Ae*. *procax*	14	33	(2/6)	17	(1/6)	50	(1/2)	0	(0/6)	0	(0/0)
*Ae*. *vigilax*	14	57	(17/30)	27	(8/30)	47	(8/17)	0	(0/30)*	0	(0/0)
*Cx*. *annulirostris*	14	0	(0/30)*	0	(0/30)*	0	(0/0)	0	(0/30)*	0	(0/0)
*Cx*. *quinquefasciatus*	14	7	(2/30)*	0	(0/30)*	0	(0/0)	0	(0/30)*	0	(0/0)
*Cx*. *sitiens*	14	0	(0/11)*	0	(0/11)*	0	(0/0)	0	(0/11)	0	(0/11)

^a^Percentage of mosquitoes containing virus in their bodies (number positive/number tested).

^b^Percentage of mosquitoes containing virus in their legs+wings (number positive/number tested).

^c^Percentage of infected mosquitoes containing virus in their legs+wings (number positive/number infected).

^d^Percentage of mosquitoes containing virus in the saliva expectorates (number positive/number tested).

^e^Percentage of mosquitoes with a disseminated infection containing virus in the saliva expectorates (number positive/number disseminated).

*Fisher’s exact test two-tailed *P*-value <0.05 for comparisons with *Ae*. *aegypti*.

### Relative quantification of ZIKV in Australian mosquitoes

**Fig 2:**
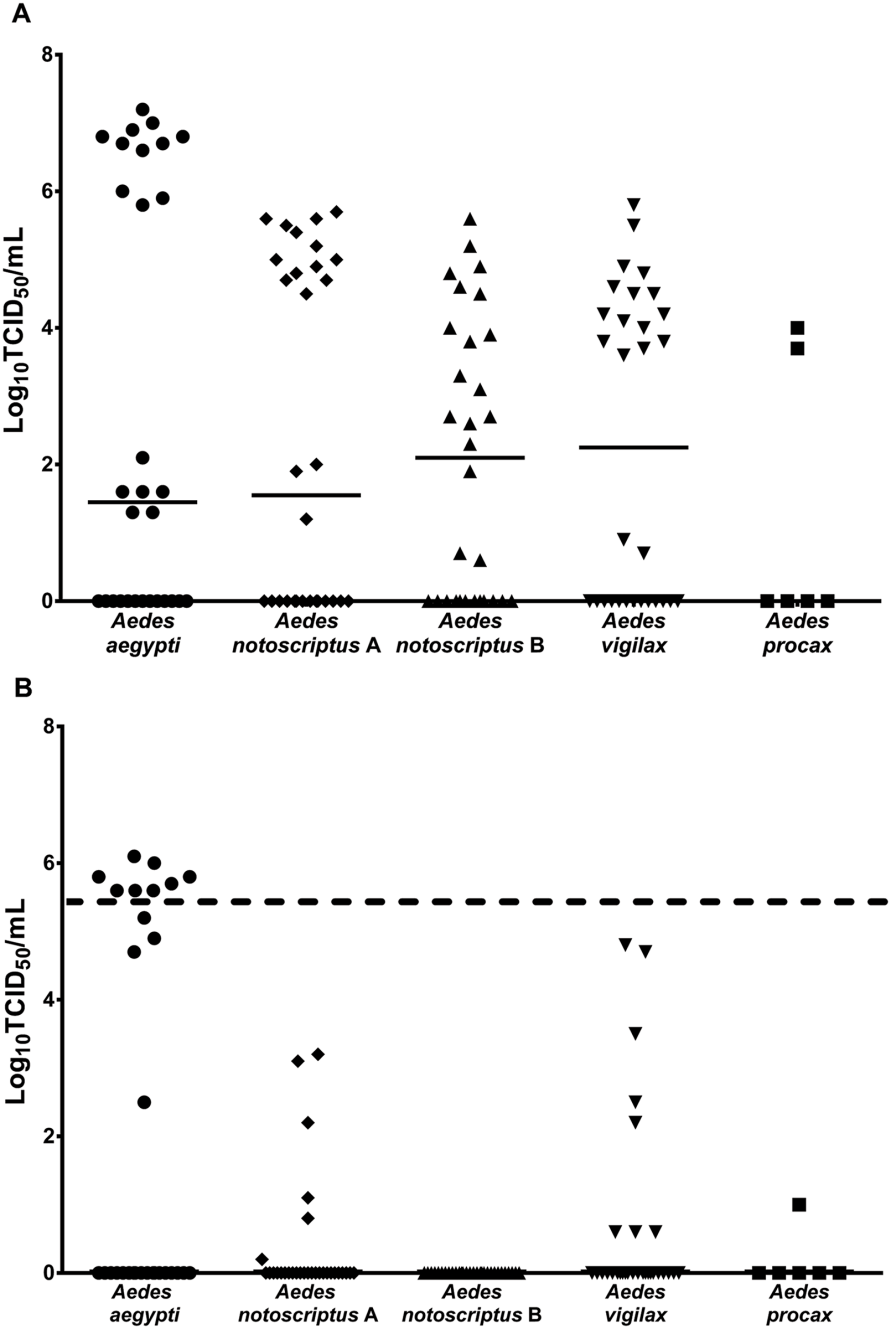
Replication of ZIKV in Australian species of *Aedes*. The titer in TCID_50_ equivalents per mL of ZIKV in the bodies (A) and legs+wings (B) of four species of *Aedes* tested 14 days after ingesting an infectious blood meal containing 10^6.7± 0.2^ TCID_50_/mL of ZIKV. *Ae*. *notoscriptus* A and B represents the two different blood feeds for this species. Each point on the plot represents an individual mosquito, and horizontal lines denote medians. *Ae*. *aegypti* had significantly higher (*P* < 0.001) legs+wings titer than *Ae*. *notoscriptus* B. The horizontal dashed line represents the threshold titer above which virus transmission occurred.

ZIKV titers in the mosquito bodies did not significantly differ among the four *Aedes* spp. tested (Fig 2). In contrast there was a significant difference (*P* = 0.0014) in the ZIKV titers in the legs+wings between these species. Importantly, transmission of ZIKV by *Ae*. *aegypti* on day 14 post exposure occurred only when the titer of ZIKV in the legs+wings was ≥ 10^5.6^ TCID_50_ equivalents/mL, with all individuals at or above this threshold able to transmit ZIKV.

**Fig 3:**
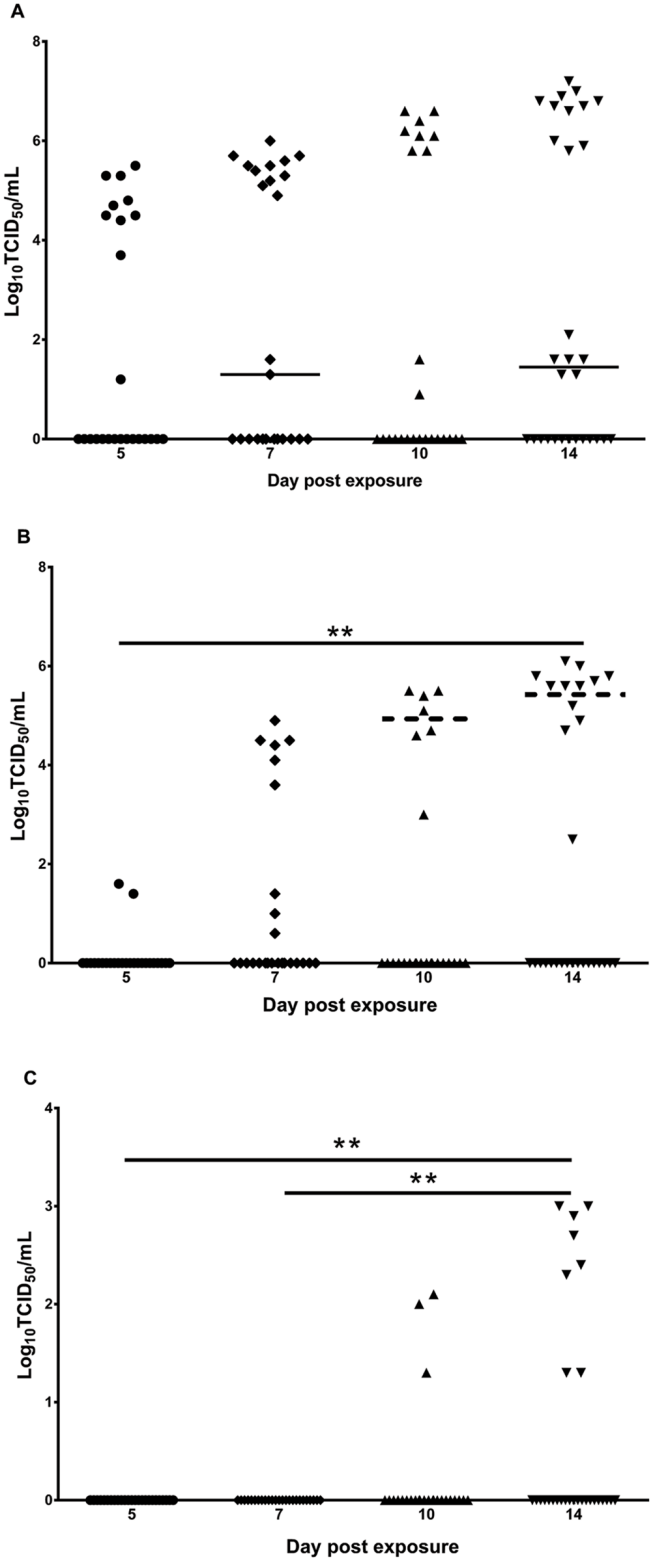
Replication of ZIKV in *Ae*. *aegypti* on different days following exposure to an infectious blood meal. The titer in TCID_50_ equivalents/mL of ZIKV in the bodies (A), legs+wings (B) and saliva (C) of *Ae*. *aegypti* and tested on different days after ingesting an infectious blood meal containing 10^6.7± 0.2^ TCID_50_/mL of ZIKV. Each point on the plot represents an individual mosquito, and bars denote medians. Solid lines represent significant differences *P* < 0.001 (**) between days for legs+wings and saliva titers. The horizontal dashed lines on days 10 and 14 represent the threshold titer above which virus transmission occurred.

For the *Ae*.*aegypti*, there was an increase in the estimated virus titer on the different days tested post exposure, although the differences were not significant (*P* > 0.05; Fig 3A). Similarly, there was an increase in legs+wings titer over the various days, with the difference between days 5 and 14 being significant (*P* < 0.05; Fig 3B). The three mosquitoes that transmitted ZIKV on day 10 post exposure possessed legs+wings titers that were lower than the 10^5.6^ TCID_50_ equivalents/mL required for transmission on day 14. The titer of the saliva expectorated did not significantly differ (*P* > 0.05) between mosquitoes sampled on days 10 and 14 post exposure (Fig 3C).

## Discussion

Commensurate with its role in transmission in Africa, the western Pacific and South America, we have demonstrated that a Townsville, Australia, population of *Ae*. *aegypti* is susceptible to infection and can transmit ZIKV. Transmission rates were higher than those recently reported for Brazilian and Senegalese strains of *Ae*. *aegypti* that were exposed to Asian and African lineages of ZIKV, respectively, but lower than those for a Singapore strain infected with MR 766 [26–28]. However, comparison of experimental vector competence outcomes between different laboratories should be viewed with caution due to differences in mosquito strain, virus strain, mosquito feeding method and virus assay used to analyze samples [29]. Regardless, we have confirmed that highly infected *Ae*. *aegypti* could be a potential source of transmission of ZIKV in Australia. Although *Ae*. *aegypti* was prevalent in eastern Australia during the first half of the 20^th^ century, the distribution of this species is currently restricted to urban areas of northern Queensland [16]. Therefore, should the virus be introduced, this is the region that is most at risk of ZIKV transmission. However, if the distribution of *Ae*. *aegypti* expanded, or if *Ae*. *albopictus*, another potential vector [26], invaded the Australian mainland, then the ZIKV receptive zone would need to be redefined. This highlights the importance of surveillance for these container-inhabiting species to ensure that any expansion of these species is recognized and elimination programs initiated.

In addition to the intrinsic ability of *Ae*. *aegypti* to transmit ZIKV, this species exhibits a number of biological traits which dramatically elevate its role as a vector of this virus, as well as other viruses transmitted by this species. Jansen et al. [30] used a relatively simple vectorial capacity model [31] to assess the relative roles of Australian mosquitoes in the transmission of CHIKV. Vectorial capacity takes into account a number of factors, including mosquito density, host feeding patterns, survival, vector competence and the duration of the intrinsic incubation period of the virus. It was demonstrated that *Ae*. *aegypti* had the highest vectorial capacity even though other species, such as *Ae*. *vigilax*, had higher experimental transmission rates [19] and population densities compared to *Ae*. *aegypti* ([32]; Queensland Health, state government data). The reason for this was that a high proportion of *Ae*. *aegypti* obtain their blood meals from humans [33, 34], and they exhibit multiple host blood feeding behavior [35], whereby females probe/feed up to four times in a single gonotrophic cycle. This latter trait exposes them to more infected humans increasing the likelihood of consuming virus, as well as increasing the number of susceptible humans exposed to an infected mosquito. The majority of other mosquito species usually take only a single blood meal per gonotrophic cycle.

To assess the potential risk for local ZIKV transmission in Australia, we exposed 6 other mosquito species to ZIKV and evaluated their ability to transmit the virus. The results demonstrated that the *Culex* spp. were either refractory to infection or exhibited a low infection rate but did not transmit ZIKV, suggesting that they should not be considered potential vectors of ZIKV in Australia. Despite being susceptible to infection, the inability of *Ae*. *notoscriptus*, *Ae*. *vigilax* and *Ae*. *procax* to transmit the virus, indicate that these species would be unlikely to play a role in ZIKV transmission. However, given the potential for intraspecies variation in vector competence of arboviruses [36, 37], it is important that the vector competence of other Australian populations of these species for ZIKV be assessed using the prototype strain, as well as the Asian lineage viral variant(s) currently circulating in the western Pacific and South America, and recently imported into Africa.

The current study has highlighted aspects of the susceptibility, replication and transmission dynamics of ZIKV in the mosquito vector that may ultimately impact transmission cycles in the field. We demonstrated that the ID_50_ for *Ae*. *aegypti* was 10^6.4^ TCID_50_/mL, which is considerably higher than that observed for other arboviruses in Australian populations of this species, including DENVs (≈ 10^5.5^ TCID_50_/mL) and CHIKV (10^4.9^ TCID_50_/mL) [19, 38]. The relatively low infection and transmission rates reported for some populations of *Ae*. *aegypti* [26, 27] would suggest a similarly high threshold for infection in this species. Paradoxically, the high ID_50_ required to infect *Ae*. *aegypti* in our study is potentially higher than the viremia values of 10^5^ to 10^6^ RNA copies per mL circulating in the blood of symptomatic patients during recent outbreaks in the western Pacific [39, 40]. This may simply reflect the differences inherent to quantifying infectious virus and viral RNA or it may hint at factors other than low susceptibility to infection of the mosquito vector. Such factors include high mosquito population density and high survival rates of infected mosquitoes, coupled with a naïve human population suffering high viral loads, and alternative modes of transmission, such as sexual transmission, all of which may contribute to epidemic transmission of ZIKV.

There are a number of intrinsic barriers that can influence the ability of an arbovirus to infect, disseminate within and be transmitted by mosquitoes [41] and the species tested in our study expressed one or more of these barriers. Based on their refractoriness to infection or low infection rate, it appears that the three species of *Culex* tested possess a midgut infection barrier. Similar to what has been observed previously with DENVs and CHIKV [19, 42], *Ae*. *notoscriptus* expresses a midgut escape barrier, as only 10% of mosquitoes tested at day 14 had a disseminated infection. With the exception of *Ae*. *aegypti*, the remaining *Aedes* spp. tested appeared to have a salivary infection/transmission barrier, as none of the individuals with a disseminated infection subsequently transmitted ZIKV. Interestingly, within the *Ae*. *aegypti* cohort, it was only those mosquitoes with a high disseminated infection titer of ≥ 10^5.1^ TCID_50_/mL in the legs+wings that transmitted the virus. This suggests that a considerable quantity of virus is required to overcome the salivary gland barriers to transmission. Conversely, the other *Aedes* spp. had lower legs+wings titers of ≤ 10^4.8^ TCID_50_/mL and this may explain why they did not transmit the virus. A correlation between the leg titer and the percentage of mosquitoes transmitting has previously been shown for dengue virus type 1 (DENV-1), whereby a threshold titer had to be reached before transmission occurred [43]. It was concluded that the high dissemination titers led to increased transmissibility by *Ae*. *aegypti*, which potentially resulted in a DENV-1 clade replacement event in Thailand. Future studies, using strains of ZIKV from the current epidemics and a considerably larger sample size, should examine whether a similar correlation exists between different viral lineages, proportion of each mosquito species infected, dissemination titers and the transmission rate. Importantly, this will reveal whether only a relatively small proportion of the mosquito population is likely to be contributing to the majority of virus transmission.

In conclusion, Australia possesses the two key elements for ZIKV to occur: a competent ZIKV vector and importation of the virus by infected travelers. The Australian distribution of *Ae*. *aegypti* is currently restricted to north Queensland, so this is the region that is receptive to ZIKV. It is paramount that container mosquito surveillance is maintained and, when resources are available, enhanced to ensure that *Ae*. *aegypti* and *Ae*. *albopictus* do not expand their range. There is also a need to maintain comprehensive testing of travelers from epidemic regions to ensure that suspected cases are diagnosed, particularly those residing in the ZIKV receptive zone. Indeed, there have already been several cases notified in northern Queensland where *Ae*. *aegypti* occurs, although control strategies implemented routinely for DENVs [44] may have assisted in preventing local transmission of the virus to date. Unfortunately, tracking of ZIKV infected travelers is compounded by the high number of asymptomatic infections and local transmission may be occurring before ZIKV is tested for in human or mosquito populations. Ultimately, a combination of case recognition, specific laboratory diagnostics, virus surveillance in mosquito populations, vector surveillance to detect incursions of potential vectors into uninfested locations and targeted mosquito control strategies will reduce the risk of an explosive ZIKV outbreak occurring in Australia.

## Supporting Information

S1 Fig Example standard curve used in each experimental run to determine viral load. The standard curve was prepared using RNA extracted from ZIKV MR 766 virus stock as template. The x-axis represents the concentration of duplicate, serially diluted template (0.01 to 10000 tissue culture infectious doses equivalents/ml) and the y-axis plots the resultant real-time RT-PCR Ct values.
(TIF)
