# Supplementary figures and images for: Assessment of Local Mosquito Species Incriminates Aedes aegypti as the Potential Vector of Zika Virus in Australia
Source: PLoS Negl Trop Dis. 2016 Sep 19;10(9):e0004959. doi: 10.1371/journal.pntd.0004959 (PMC5028067; doi:10.1371/journal.pntd.0004959)

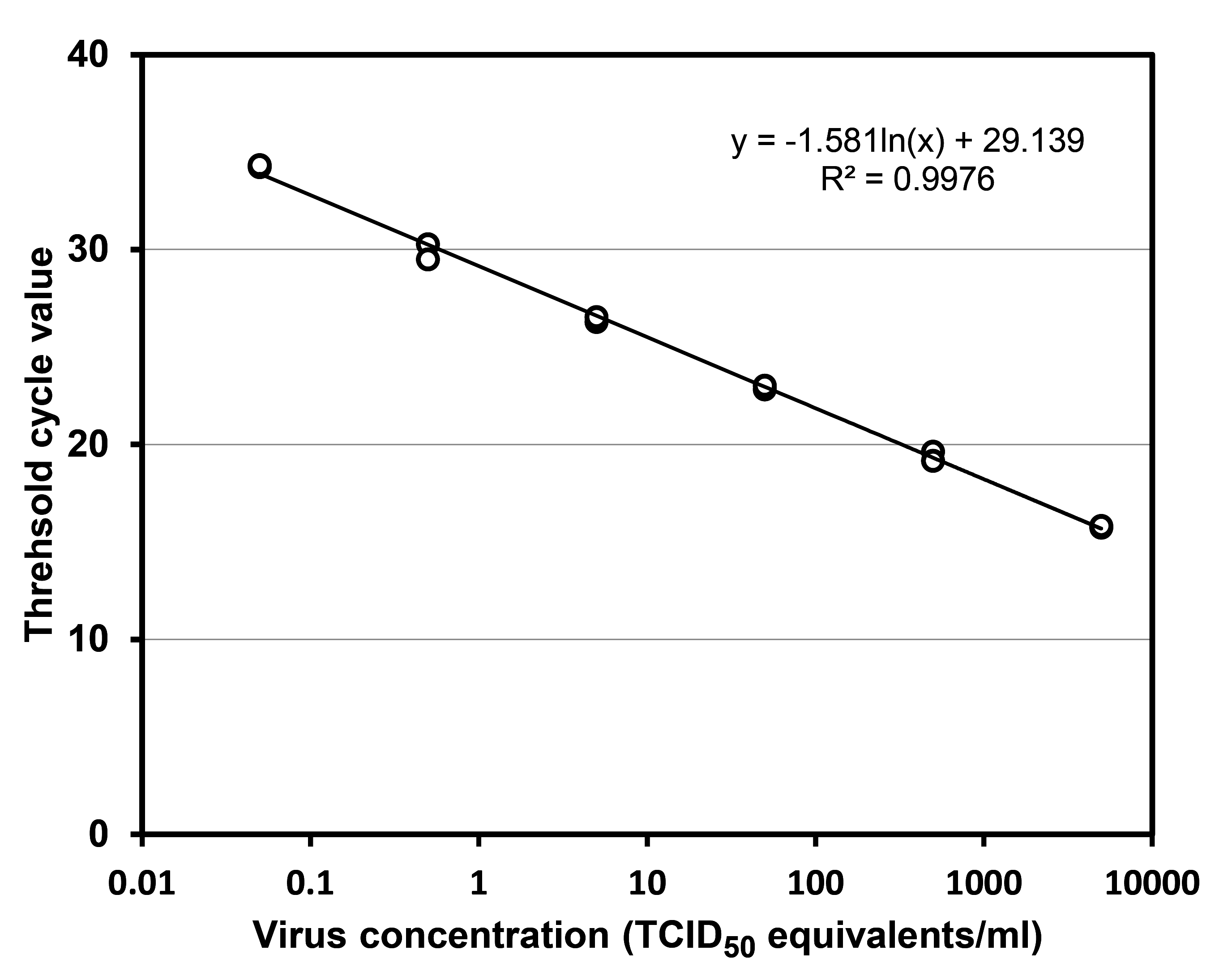

Supplement: S1 Fig — The standard curve was prepared using RNA extracted from ZIKV MR 766 virus stock as template. The x-axis represents the concentration of duplicate, serially diluted template (0.01 to 10000 tissue culture infectious doses equivalents/ml) and the y-axis plots the resultant real-time RT-PCR Ct values. (TIF) [file pntd.0004959.s001.tif]
